# Medical Malpractice in Neurosurgery: An Analysis of Claims in the Netherlands

**DOI:** 10.1227/neu.0000000000003117

**Published:** 2024-07-26

**Authors:** Wouter J. Dronkers, Dennis R. Buis, Quirine J. M. A. Amelink, Gert-Joan Bouma, Wilco C. Peul, W. Peter Vandertop, Marike L. D. Broekman, Aart C. Hendriks, Clemens M. F. Dirven, Jochem K. H. Spoor

**Affiliations:** * Department of Neurosurgery, Erasmus University Medical Centre Rotterdam, Rotterdam, The Netherlands;; ‡ Department of Neurosurgery, Amsterdam University Medical Centre, University of Amsterdam, Amsterdam, The Netherlands;; § Amsterdam Neuroscience Centre, Neurovascular Disease, Amsterdam, The Netherlands;; ‖ Department of Legal Affairs, The Dutch Health and Youth Care Inspectorate, Utrecht, The Netherlands;; ¶ Erasmus University Rotterdam, Erasmus School of Health Policy and Management, Rotterdam, The Netherlands;; # Department of Neurosurgery, Leiden University Medical Center, Leiden, The Netherlands;; ** Department of Neurosurgery, Haaglanden Medical Center, The Hague, The Netherlands;; ‡‡ Faculty of Law, Leiden University School of Law, Leiden, The Netherlands

**Keywords:** Claim, Health law, Malpractice, Neurosurgery, Spine surgery

## Abstract

**BACKGROUND AND OBJECTIVES:**

Studying malpractice claims is important to improve quality of health care and patient safety and to educate the individual healthcare providers. The objective of this study was to describe characteristics of neurosurgical claims in the Netherlands.

**METHODS:**

A nationwide retrospective observational study of neurosurgery-related claims closed by Centramed and MediRisk, 2 major insurance companies in the Netherlands, was performed. Relevant data, including type of neurosurgical pathology, theme and category of the claim, type and severity of injury, outcome, and financial burden, were extracted from anonymized claim files. The estimated annual risk was used to determine the risk for claims by adjusting for the number of annually practicing neurosurgeons in the Netherlands.

**RESULTS:**

A total of 388 claims against neurosurgeons were closed between 2007 and 2021. Liability was denied in a slight majority of claims (n = 230; 59%). The total burden during this period was €6 165 000 (amount paid out to patients: €5 497 000). The estimated annual risk per Dutch neurosurgeon for a claim was 15.5%, meaning 1 claim per 6.5 years. The case-level analysis of 238 available anonymized claims revealed that most claims were related to spinal pathology (81.5%), followed by cranial pathology (10.9%) and peripheral nerve (7.6%). The motivations for filing claims were mostly related to alleged surgical (56.3%) or diagnostic errors (22.3%). Most of these claims were denied (151/238; 63.4%), and fewer were settled (42/238; 17.6%), sustained (31/238; 13.0%), or closed without final decision (14/238; 5.9%).

**CONCLUSION:**

Neurosurgery-related malpractice claims primarily involved spinal pathology and were mostly related to alleged treatment errors. Most claims did not result in compensation because there seemed to be no liability or culpable injury. However, the annual risk for a claim for Dutch neurosurgeons is considerable.

**ABBREVIATIONS:**
CUO: claim with an unfavorable outcome
DTC: Diagnosis-Treatment Combinations
EAR: estimated annual risk.

Neurosurgeons are the physicians who get many malpractice claims.^1-6^ Studying neurosurgical claims may be important because evaluation of these claims may add to improve the quality of neurosurgical care and patient safety and to educate the individual neurosurgeon. This study aims to determine the characteristics of neurosurgery-related malpractice claims in the Netherlands.

## METHODS

### Legal Background

The Netherlands does not have a so-called no-fault insurance system. A detailed overview of the Dutch legal system including various legal routes, an elaboration on medical malpractice, and assessment of liability is provided in **Supplemental Digital Content 1** (http://links.lww.com/NEU/E398).

### Data Source

In the Netherlands, medical specialists of a particular hospital are collectively insured through a specific (malpractice) insurance company. This is contrary to other countries in which physicians are individually insured through its own insurance company. Eighty-five percent of all Dutch hospitals have their malpractice insurance covered at 2 insurance companies, Centramed and MediRisk. These 2 major insurers provided data for this study. Permission was requested from the board of insurers to both obtain and analyze anonymized claim data. Limited time access to claim files was granted to first and senior authors. These claim files contained liability statement, rebuttal and rejoinder, relevant copies of the patients' chart, independent injury assessment by a third party, liability assessment, correspondence between the parties, and concluding statement by the insurer on the outcome. Individual participant informed consent was not applicable, and ethical board review was waived for this study (METC 2020-0972).

### Data Collection Process and Parameters

All neurosurgical claims closed between January 1, 2007, and December 31, 2021, were included for exploring trends and to calculate the estimated annual risk (EAR) for the individual neurosurgeon. Furthermore, anonymized closed claims between 2007 and 2017 that were available for in-depth case-level analysis were studied for characteristics. Most of the claim files after 2018 are not yet anonymized and could therefore not be studied in depth.

Centramed data were obtained through a data collection process at its facility using paper files. MediRisk data were obtained from RStudio IDE and Azure Data Studio (Microsoft Azure). Patient names, physician names, hospital names, and other privacy sensitive data were anonymized by the insurance companies before the data collection. The first author extracted the data from all closed claims manually. Thereafter, the first- and senior author rereviewed 10% of all claims together to ensure data integrity.

Demographic data for plaintiff characteristics (age category), defendant characteristics (attending or resident physician), and neurosurgical subspecialty (cranial, spinal, peripheral nerve, and pediatric neurosurgery) were collected. Clinical data that were collected involved: primary diagnosis, neurosurgical procedure and additional treatment, and adverse events.

### Complaint Categories and Type and Severity of Injury

The claim could contain more than 1 complaint. Therefore, these complaints were stratified in predefined categories, which were developed by the authors (**Supplemental Digital Content 2**, http://links.lww.com/NEU/E399). Six complaint categories emerged: surgical/technical error, diagnostic error or delay in treatment, insufficient informed consent or improper indication of treatment, insufficient care in general (during hospital stay or follow-up), communication, and other.

The type and severity of injuries were assessed according to a predefined classification system (**Supplemental Digital Content 2**, http://links.lww.com/NEU/E399). Categories of injuries involved: physical harm (subcategories: persisting pain, increased or new sensory-motor deficits, other [organ damage, cosmetic issues], and death), emotional harm, and financial loss. Patients could have multiple types of injuries. For severity, 6 categories were used: minor temporary, minor permanent, major temporary, major permanent, catastrophic, and death.

### Study Outcome

Outcomes involved the claim volume (absolute number of closed claims) and financial burden. These outcomes were reported in total and per neurosurgical subspecialty. For this, cranial subspecialties were subsequently stratified into tumor, vascular, functional, trauma, hydrocephalus, and infectious. Spine surgery was subsequently stratified into degenerative, tumor, trauma, and other (eg, infections).

### Statistical Analysis

Continuous variables are provided as means ± SDs, and categorical variables as numbers (percentages). Descriptive statistics were performed for all variables. For outcome, claims denied or closed without decision were considered “favorable for the neurosurgeon.” Claims sustained or settled were considered “unfavorable for the neurosurgeon.” The EAR was calculated by dividing the number of claims by the annual number of registered neurosurgeons multiplied by the number of studied years (15 years). All analyses were performed using IBM SPSS, version 28 (IBM Corp). *P*-values <.05 were considered statistically significant on two-tailed tests. The total cost of claims was reported in Euros (€).

## RESULTS

### Neurosurgical Volume and Number of Registered Neurosurgeons

Diagnosis-Treatment Combinations (DTCs) used for medical billing and reimbursements are centrally registered by the Centraal Bureau voor de Statistieken (Central Bureau for Statistics), an official Governmental body.^7^ A DTC holds entities related to a specific disease. For example, within the DTC for “spinal stenosis,” several entities (outpatient consultation, diagnostics, surgical procedure) are incorporated. In most, but not all cases, a surgical procedure will take place. For example, the DTC “cervical fracture” may hold a conservative treatment course with a collar but could eventually hold a surgical procedure. The DTCs serve as an estimate to determine the ratio between spine and cranial procedures. Annually, approximately 75 000 neurosurgical DTCs (44 000 spine, 19 000 cranial, and 13 000 peripheral nerve) were filed from 2007 up to 2021. These findings resulted in a spine to cranial DTC ratio of 1: 0.43 and a spine to peripheral nerve DTC ratio of 1: 0.29. DTC data on pediatric neurosurgery could not be retrieved. On average, approximately 150 neurosurgeons were practicing in the Netherlands during this period, covering approximately 17.9 million citizens.^8^

### Trends in Filing and Closing (Period: 2007-2021)

An upward trend for filed neurosurgical claims can be noticed between 2007 and 2017 with a decrease in filed claims from 2018 onward compared with the years before (Figure). Between 2007 and 2021, a total of 18 649 claims against physicians in hospitals were closed by Centramed and MediRisk.^9^ Three hundred eighty-eight (2.1%) claims were against a neurosurgeon or a neurosurgical resident. Most claims (n = 230; 59%) were denied (56 [15%] sustained, 78 [20%] settled, 24 [6%] closed without a final decision) (Table 1).

**FIGURE.**
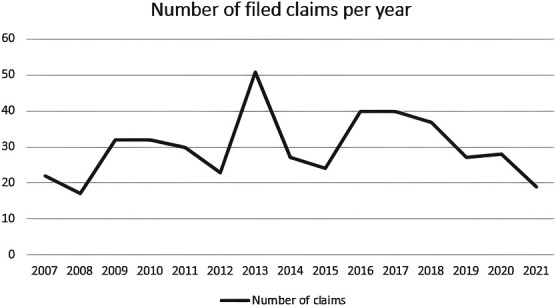
An overview of the number of claims filed per year.

**TABLE 1.** Trends of Closed Malpractice Claims (2007-2021)

Claim volume	
Filed	449 (100)
Closed	388 (86)
Outcome (closed claims)	
Denied	230 (59)
Sustained	56 (15)
Settled	78 (20)
Closed without final decision	24 (6)
Financial burden	
Total burden	€6 165 000
Total patient pay-out	€5 497 000
Median burden CUO (IQR)	€16 700 (€43 000)
Median patient pay-out CUO (IQR)	€10 000 (€30 900)
Risk for malpractice	
EAR overall	15.5%
Interpretation overall	One claim every 6.5 years
EAR CUO	5.4%
Interpretation CUO	One claim every 18.7 years

CUO, claim with an unfavorable outcome; EAR, estimated annual risk.

The total financial burden for all closed neurosurgical claims was €6 165 000, of which €5 497 000 (89.2%) was paid out to patients. The median (IQR) burden per claim with an unfavorable outcome was €16 700 (€43 000) with a median (IQR) pay-out per patient of €10 000 (€30 900).

The EAR for a claim, based on the number of closed claims, was 15.5%. This risk can be interpreted as 1 claim every 6.5 years. The risk for a claim with an unfavorable outcome (CUO) (sustained or settled) was 5.4%, interpreted as 1 CUO every 18.7 years.

### Case-Level Analysis (Period: 2007-2017)

A total of 238 claims, closed between 2007 and 2017, were available for in-depth analysis (Table 2). Claims mostly involved spine surgery (n = 194; 81.5%), followed by cranial surgery (n = 26; 10.9%) and peripheral nerve surgery (n = 18; 7.6%). Most claims involved elective cases (n = 218; 91.6%). Often, incidents that led to a claim took place during the perioperative stage in the operating room (n = 146; 61.3%).

**TABLE 2.** Case-Level Analysis Claims (2007-2017)

General characteristics	
Claim volume, closed claims	238 (100)
Career stage defendant, consultant neurosurgeon	229 (96.2)
Type of neurosurgery	
Spine surgery	194 (81.5)
Degenerative	177 (74.4)
Tumor	2 (<1)
Trauma	3 (1.3)
Other	4 (1.7)
Not specified	8 (3.4)
Cranial surgery	26 (10.9)
Tumor	13 (5.5)
Hydrocephalus	5 (2.1)
Functional	4 (1.7)
Vascular	3 (1.3)
Trauma	4 (1.7)
Peripheral nerve surgery	18 (7.6)
Carpal Tunnel Syndrome	9 (3.8)
Other nontumor	6 (2.5)
Tumor	3 (1.3)
Pediatric neurosurgery	1 (<1)
Care characteristics	
Urgency of care, elective cases	218 (91.6)
Location of the primary incident	
Operating room	147 (61.8)
Ward (including ICU)	42 (17.6)
Outpatient setting	40 (16.8)
Emergency department	3 (1.3)
Location not specified	9 (2.5)
Stage of care	
Preoperative	43 (18.1)
Perioperative	146 (61.3)
Postoperative	34 (14.3)
Postdischarge	5 (2.1)
Stage not specified	10 (4.2)

ICU, Intensive Care Unit.

Motives for filing a claim mostly involved alleged surgical or technical errors (n = 134; 56.3%), diagnostic errors or delay in treatment (n = 53; 22.3%), and insufficient informed consent or treatment indication (n = 21; 8.8%) (Table 3). Liability was denied in most of the claims for spine-, cranial-, and peripheral nerve–related claims (Table 4). Persisting pain constitutes a contributing factor in 121/238 (50.8%) claims.

**TABLE 3.** Motivation for a Claim and Injuries per Type of Neurosurgery (2007-2017)

	Total	Spine	Cranial	Peripheral nerve
Motivation, (*alleged…*)				
Surgical/technical error	134	111	11	12
Diagnostic error or delay in treatment	53	40	8	5
Insufficient informed consent or indication for treatment	21	19	1	1
Insufficient care				
During hospital admission	12	11	1	—
Post-discharge/follow-up	7	4	3	—
Communication	6	4	2	—
Other	1	1	—	—
Not specified	4	4	—	—
Injury, *type*				
Physical harm	212	180	16	2
Persisting pain	121	109	0	9
Increased or new sensory- and/or motor deficits	59	50	5	7
Other (eg, loss of organ function, cosmetic damage)	24	19	5	—
Death	8	2	6	—
Emotional harm	47	32	9	6
Financial loss	33	29	2	2
Injury, *severity*				
No injury	3	2	1	—
Minor temporary	13	11	1	1
Minor permanent	132	117	11	4
Major temporary	3	2	—	1
Major permanent	71	54	5	12
Catastrophic	3	2	1	—
Death	8	6	2	—
Not specified	5	4	1	—

**TABLE 4.** Outcome per Type of Neurosurgery (n = 238 Claims; 2007-2017)

Type of neurosurgery	Volume	Outcome				Financial burden	Median burden per claim
		Denied	Sustained	Settled	No decision		
Spine	194	127	25	29	13	M2,3 Euro	K11,8 Euro
Degenerative	177/194	118	21	27	11		
Cranial	26	11	4	10	1	K400 Euro	K16,6 Euro
Tumor	13/26	6	4	3	—		
Hydrocephalus	5/26	2	—	2	1		
Functional	4/26	3	—	1	—		
Trauma	4/26	1	1	2	—		
Vascular	3/26	1	—	2	—		
Peripheral nerve	18	13	2	3	—	K210 Euro	K11,6 Euro
Total	238	151	31	42	14	M2,9 Euro	K12,1 Euro

## DISCUSSION

We studied the characteristics and the EAR for malpractice claims in neurosurgery over a period of 15 years in the Netherlands. Neurosurgeons face an EAR for a claim of 15.5% (on average 1 claim every 6.5 years). In most claims, liability was denied, resulting in an EAR for a CUO (sustained or settled claims) of 5.4%. Studying 238 claims on a case-level revealed that most of the claims involved elective, degenerative spine care, mostly involving alleged perioperative surgical errors resulting in nonrelief of pain.

An upward trend regarding filed neurosurgical claims since 2007 in the Netherlands is noticeable, with a peak in 2013. A decrease in claims is noticeable from 2018 onward. Partially, this decrease in the number of filed claims could be the due to the COVID-19 pandemic. During the pandemic, elective care was largely deferred because of scarcity in hospital beds, personnel, and other resources. In this study, most claims were related to elective spinal care. A decrease in the absolute number of (spinal) surgeries might have therefore resulted in fewer claims in those particular years. Future studies may study the trends from 2021 onward to conclude upon this hypothesis. Previous research on neurosurgery-related claims is mostly conducted in the United States and the United Kingdom,^10-13^ with fewer studies conducted in Europe and other continents.^14,15^ Comparing present findings with previous studies conducted in the United States and the United Kingdom reveals similarities and differences. Similar to these studies, we found an over-representation of spine-related claims. Furthermore, most of the claims were filed because of alleged diagnostic- and treatment-related incidents, similar to our findings. A major difference to be noted is related to the height of the financial burden. In the United States and United Kingdom, it is not unlikely for neurosurgical claims to exceed a monetary value of 1 million Dollars or Pounds per claim.^11,13^ Patient payouts in sustained and settled claims were considerably lower in the Netherlands. One explanation may be the influence of the Dutch government, acting as the welfare state providing financial support for citizens in the case of protracted illness, injury, and disability. Physicians causing damage to patients are therefore, to large extent, not responsible for reimbursement of expenses such as additional treatments, adjustments to homes and transportation in the case of disability, or compensating loss of income.

Spine-related care has been previously reported to be a risk factor for both neurosurgeons and orthopedic surgeons.^16-18^ In this study, we found that almost 3 quarters of all claims that were studied on a case-level involved elective, degenerative spinal surgery. At least 2 reasons could explain the relatively high number of claims after spine care. First, the surgical volume of spine cases was compared with that of cranial and peripheral nerve cases. Spinal surgery, especially degenerative spine care, is often regarded as a major part of the daily practice for many neurosurgeons in the Netherlands. Being more exposed to spine patients will intrinsically result in an increased risk for a claim. Therefore, we determined the ratio between spine, cranial, and peripheral nerve care by adjusting for volume of care. Based on the closed billing codes, used for reimbursements, we determined a ratio of 1 spine case per 0.43 cranial case. Based on the ratio between degenerative spine (177 claims) and cranial (26 claims) cases, it can be concluded that factors other than absolute volume of care influence the increased risk for a claim during spine care. Management of patients' expectations is important, especially before and during elective treatment. In this regard, expectation management is closely related to the shared decision-making process and informed consent. Studying claims on a case-based level, we found that a substantial number of spine care claims were filed because of the lack of perceived treatment effect, resulting in persisting or increased pain complaints. Addressing aspects such as the expected outcome of a certain treatment remains vital. In this study, particularly in spine-related claims, expectation management might have fallen short when considering the number of claims filed because of either a lack of pain relief or a lack of informed consent. A recommendation that follows from these findings involves a re-evaluation of one's practice regarding the informed consent process. This process does not only involve educating patients on possible adverse events but should also entail the indication and the possibility of not reaching the expected results despite surgery. Persisting (leg and/or back) pain remained an important issue in spine-related claims. Adjusting one's counseling may contribute to lowering one's risk for claims in the future.

Legal interest in medical malpractice and “claim culture” may also, to some extent, explain the difference in the number of malpractice claims and the risk for litigation. For example, Japanese neurosurgeons seem to be less prone for a claim with only 95 closed claims between 1961 and 2017, against over 3800 active Japanese neurosurgeons.^16^ Contrarily, 2131 claims were closed in the United States between 2003 and 2012 against over 7155 board-certified neurosurgeons in the United States.^10,19^ It should be noted that studies often reported on a fraction of the total number of claims and therefore lack overall representability for a country or solely report on particular subspecialties and/or a particular disease.^10,16,20-25^ For estimating the risk for a claim, it is necessary to adjust for specialty size and “completeness” of data. In this study, we used the number of registered neurosurgeons to estimate the annual risk in the Netherlands by pooling the number of claims of the 2 main malpractice insurers who cover over 85% of all Dutch hospitals.

Risk assessment is important for physician education, and the (emotional) impact of patients' complaints and claims should not be underestimated, even if the claim or complaint is denied. Physicians who are prone to receiving complaints and claims are more likely to report increased levels of stress, feelings of anxiety and depression, and burnout.^26,27^ In our study, we found an EAR of 15.5% (about 1 claim every 6.5 years) for neurosurgeons practicing in the Netherlands, which is slightly lower than the estimated risk of 19.1% US neurosurgeons faced but still substantial.^28^ Because liability was denied in most claims or claims were closed without a verdict, the actual risk for a CUO was 5.4%, which may provide a more nuanced perspective. Regardless of the outcome, receiving a claim might have an impact on the neurosurgeons' well-being. Therefore, it is important to create an open culture with peer support when dealing with patients' complaints and claims.

This study holds some limitations. First, not all claims between 2007 and 2021 were available for case-level analysis because the anonymization process that these claims have to go through to fulfill the requirements of the Dutch General Data Protection Regulation was incomplete as a consequence of new COVID-19 regulations. Furthermore, because of this regulation, we were not able to determine the number of neurosurgeons who might have been responsible for a disproportionate number of claims. This may be relevant when taking the EAR into account. However, the aim of this study was to provide a novel method to give estimated risks. By no means, this study aimed to give certain neurosurgeons who might have been responsible for a disproportionate number of claims the feeling of “calling out” upon the alleged lack of care they provided.

This may to some extent introduce bias in the presented data because not all closed cases were available for analyses. Despite the fact that not all claims were studied on a case-level basis, we think that a 10-year period from 2007 to 2017 provided a sufficient number of claims to study the characteristics of neurosurgical claims. The retrospective nature, which is inevitably related to research on malpractice data, comes with limitations. Unfortunately, data on malpractice cannot provide real-time information about the current situation within a particular country or medical specialty. Nevertheless, much of the insights that can be drawn from these findings are still applicable in today's practices. Finally, it is important to keep in mind that claims only represent the “tip of the iceberg” of incidents that occur on a daily basis. Future studies may address the perception of neurosurgeons toward malpractice litigation and the perceived stress when confronted with claims and complaints.

## CONCLUSION

Malpractice claims related to neurosurgery primarily involve degenerative spinal pathology and are mostly related to alleged treatment errors. Persisting pain constitutes a contributing factor in these claims. Neurosurgeons may re-evaluate their practice of patients' expectation management and informed consent process to both better help patients and minimize the risk for malpractice litigation in the future. Most claims did not result in compensation because there seemed to be no liability or culpable injury. Regardless, the annual risk for a malpractice claim for Dutch neurosurgeons is substantial.

## Supplementary Material

## SUPPLEMENTAL DIGITAL CONTENT

**Supplemental Digital Content 1. Table 1.** A legal overview of the Dutch legal system for healthcare-related issues. **Supplemental Digital Content 2. Table 2.** Examples of types of claims and injuries.

## COMMENTS

The take-home lessons from this study of neurosurgical medical malpractice in the Netherlands are as follows:

Neurosurgeons in the Netherlands have an average 15% chance per year of having a malpractice claim made against them, only marginally less than the 19%/year chance neurosurgeons have in the United States. A litigious social culture may explain much of the difference in frequency of malpractice claims among different nations. The authors point out that in the 56 years between 1961 and 2017, Japanese neurosurgeons had only 95 closed claims, ie, less than 1 per year. It cannot be just a difference in surgical proficiency.

Spinal surgery carries a disproportionately high malpractice risk for neurosurgeons. Why? Perhaps because of unrealistically high expectations of success measured as pain relief by both the surgeon and the patient, perhaps because of surgery performed for marginal surgical indications, perhaps because of poor communication and inter-relation between the surgeon and the patient. The reasons are speculative, but the phenomenon is not unique to the Netherlands: neurosurgeon malpractice studies in the United States reveal a similar predominance of spinal surgery among malpractice claims.

A generously tax-funded social welfare system that covers medical bills and provides salary income substitutes for the injured and disabled is not the primary reason for differences in medical malpractice claims among different countries. Workers’ compensation insurance in the United States provides medical expense coverage and income substitute for injured workers, but malpractice claims are not proportionately reduced among workers’ compensation patients. There is something else propelling that boat.

The authors reason that the study of malpractice claims may improve quality and safety of care and better educate neurosurgeons on how to avoid medical liability. In 2004, David Studdert wrote in the New England Journal of Medicine that, “several factors have been linked to patients' decisions to bring malpractice claims, most notable patient dissatisfaction and physicians' communication and interpersonal skills.”^1a^ Perhaps the best use of this study is to prompt neurosurgeons to examine themselves introspectively for their degree and quality of empathy rather than searching among objective surgical technical details or patient motives for the causes of their medical liability claims.

**James R. Bean**

*Lexington, Kentucky, USA*

## Notes

The authors analyzed neurosurgical malpractice claims in Holland over a 14-year period. While most claims were denied, nearly a third of them ended with a settlement or a judgment against the defendant neurosurgeon—ie, “adversely.” They estimated the annual “risk” of a claim for practicing neurosurgeons in Holland to be about 15%—not a low number. As in the United States, claims related to spine surgery were the most common, and surgical/technical errors were the basis for most claims, as opposed to deficiencies in communication (at least as far as could be gleaned from the records).

There had been a trend starting in 2013 for claims to increase, and the subsequent decrease was logically attributed to the effects of the COVID-19 pandemic and the temporary halt in elective surgery. Perhaps that hiatus may have led to a reset in attitudes in the Netherlands away from a litigious culture that may be an export from the United States, along with popular entertainment of all kinds.

American neurosurgeons, who have been medicolegally looking over their shoulders for decades, may take some small measure of comfort in the shared misery of their Dutch colleagues. Perhaps we can learn from their healthcare system how to improve population insurance coverage as a way to reduce the size of malpractice payouts, but that is a bigger topic for another day.

**Michael Schulder**

*Manhasset, New York, USA*
