# Supplementary material for: Medical Malpractice in Neurosurgery: An Analysis of Claims in the Netherlands
Source: Neurosurgery. 2024 Jul 26;96(3):673–80. doi: 10.1227/neu.0000000000003117 (PMC11789863; doi:10.1227/neu.0000000000003117)
Supplement: Supplementary file 1 [file neu-96-673-s001.docx]

**Supplement table 1.** Legal Background

| The Netherlands does not have a so-called no-fault insurance system. Patients that want to be eligible for financial compensation for alleged medical malpractice need to submit a claim. There are several legal procedures patients can make use of to submit such a claim, depending on the aim of the procedure. These legal routes have different aims and can be initiated simultaneously. It is important to note that not all complaints result in the initiation of formal litigation. The two main routes involve a disciplinary procedure and a medical malpractice procedure. The Dutch Disciplinary Law aims to increase the quality of care and patient safety. Issues that are commonly handled through disciplinary law involve professionalism and communication. Previous research suggests that the absolute risk for a complaint through disciplinary law in the Netherlands is low for neurosurgeons^7^. Disciplinary complaints against Neurosurgeons were largely related to preoperative care, with a large proportion of complaints related to informed consent and communication issues. Medical malpractice procedures are on the basis of civil law standards in an out-of-court setting. This commonly involves damage for which compensation is sought. Damage can be physical, emotional, financial (e.g., loss of income), or a combination of these types. Most often, a malpractice case is initiated through a complaint with a request for compensation addressed against the physician through a hospital complaints officer. Fewer cases are initiated through an independent complaints dispute or are filed directly at a civil court. Assessment of liability is mostly done by a malpractice insurance company which handles the claims for a particular hospital through which physicians are insured. Damage emerging prior-, during or after the provision of care does not automatically lead to the assumption of liability. For liability, five cumulative criteria have to be met. The first criterion is that there must be an incident. The second criterion is that there must be negligence. The third criterion is that the negligence can be attributed to the actions or omissions of a caregiver or healthcare institute relative to a certain norm or protocol. The fourth criterion holds that in principle, the claimant has to prove the actual damage. Finally, the claimant has to prove that the actual damage is caused by the actions or omissions of the caregiver or healthcare institute (causal effect), which can be attributed to supplied interventions outside the regular way of handling care. Proof of negligence is assessed by a medical expert (neurosurgeon) who is contracted by the insurer. However, parties can agree upon an independent medical expert who is not affiliated with the insurer. |
| --- |
