# Supplementary material for: Medical Malpractice in Neurosurgery: An Analysis of Claims in the Netherlands
Source: Neurosurgery. 2024 Jul 26;96(3):673–80. doi: 10.1227/neu.0000000000003117 (PMC11789863; doi:10.1227/neu.0000000000003117)
Supplement: Supplementary file 2 [file neu-96-673-s002.docx]

**Supplement table 2.** Motivation for claim (examples) & Type and Severity of Injuries

| **Motivation for claim;** alleged… | **Examples** (non-exhaustive) |
| --- | --- |
| Surgical/technical error | *Wrong surgical approach; wrong level/ -side surgery; perioperative nerve damage; pedicle screw malposition; inadequate positioning of the patient* |
| Diagnostic error or delay in treatment | *Wrong diagnosis; late diagnose due to lack or inadequate use of diagnostics; delay in recognition of cauda syndrome; referral delay* |
| Insufficient informed consent or improper indication for treatment | *Lack or absence of informed consent (or parts of it); unjustified surgery or wrong indication for spine surgery* |
| Insufficient care |  |
| During hospital admission | *Insufficient care during hospital admission other than diagnostics; medication errors; inadequate treatment of postoperative infections* |
| Post-discharge/follow-up | *No- or lack of follow-up* |
| Communication | *Lack or inadequate intercollegiate communication; lack or inadequate communication with patient other than informed consent* |
| Other |  |
| **Type of injuries** |  |
| Physical harm | *New or persisting neurological deficits (e.g., paraplegia); loss of function (e.g., loss of bladder function); persisting pain* |
| Emotional harm | *Increased stress, anxiety or grief* |
| Financial loss | *Incapacitated or not being able to work to the same extent as before the incident* |
| **Severity of injuries** |  |
| Minor temporary | *Wound infection, transient pain, stress due to unplanned re-surgery* |
| Minor permanent | *Isolated paresis of a limb, persisting pain, cosmetic complaints; inability to work; vocal cord paresis* |
| Major temporary | *Transient physical dysfunctions (e.g., bladder, hand); damage to prosthesis (e.g., breast prosthesis)* |
| Major permanent | *Complete and partial thoracic level spinal cord injuries; partial cervical spinal cord injuries; combination of three or more minor permanent injuries (e.g., isolated paresis + persisting pain & bladder dysfunction); damage to internal organs (e.g., intestines, liver)* |
| Catastrophic | *Complete cervical spinal cord injuries; wrong diagnosis resulting in untreated metastasis* |
| Death | - |
